# Discordant Correlation between Serological Assays Observed When Measuring Heterosubtypic Responses against Avian Influenza H5 and H7 Viruses in Unexposed Individuals

**DOI:** 10.1155/2014/231365

**Published:** 2014-06-11

**Authors:** Eleonora Molesti, Francesca Ferrara, Giulia Lapini, Emanuele Montomoli, Nigel Temperton

**Affiliations:** ^1^Viral Pseudotype Unit, Medway School of Pharmacy, University of Kent, Central Avenue, Chatham Maritime, Chatham ME4 4TB, UK; ^2^Department of Molecular and Developmental Medicine, University of Siena, Via Aldo Moro 3, 53100 Siena, Italy

## Abstract

The human population is constantly exposed to multiple influenza A subtypes due to zoonotic spillover and rapid viral evolution driven by intrinsic error-prone replication and immunological pressure. In this context, antibody responses directed against the HA protein are of importance since they have been shown to correlate with protective immunity. Serological techniques, detecting these responses, play a critical role for influenza surveillance, vaccine development, and assessment. As the recent human pandemics and avian influenza outbreaks have demonstrated, there is an urgent need to be better prepared to assess the contribution of the antibody response to protection against newly emerged viruses and to evaluate the extent of preexisting heterosubtypic immunity in populations. In this study, 68 serum samples collected from the Italian population between 1992 and 2007 were found to be positive for antibodies against H5N1 as determined by single radial hemolysis (SRH), but most were negative when evaluated using haemagglutination inhibition (HI) and microneutralisation (MN) assays. As a result of these discordant serological findings, the increased sensitivity of lentiviral pseudotypes was exploited in pseudotype-based neutralisation (pp-NT) assays and the results obtained provide further insight into the complex nature of humoral immunity against influenza A viruses.

## 1. Introduction

The constant rapid evolution of HPAI H5 and H7 viruses driven by intrinsic error-prone replication and increased by immune pressure significantly influences the sensitivity of available serological assays. Moreover, the antigenic variation of influenza viruses can also limit the efficacy of prepandemic human vaccines, vaccine strain selection, and results in the necessity to update influenza vaccines to include contemporary viruses and to monitor those that are distinct from the current vaccine strains [1]. The human population is constantly exposed, during a lifetime (by natural infection and/or vaccination) to different influenza A subtypes with an associated increase in the memory B cell repertoire. This antibody repertoire may also be cross-reactive to closely related variant viruses making it more difficult to develop sensitive and specific serological assays [2–4]. The adaptive homosubtypic antibody responses to the antigenic sites of the HA and NA of individual influenza strains can discriminate between influenza subtypes and current seasonal influenza vaccines can boost strain-specific responses with little protection against antigenically drifted or shifted strains. However, it has been shown that exposure to one subtype of influenza A can also induce immunity that is cross-protective against other influenza subtypes. Such adaptive immune response, called “heterosubtypic” immunity, elicits an antibody response to epitopes that are highly conserved amongst strains [5–7]. These more conserved epitopes, which are less accessible than those on the HA globular head, are predominantly localized in the membrane-proximal stalk region of HA [8]. From 2009 until recently, many anti-stalk antibodies have been structurally analyzed [9–12] and increased attention has been focused to understand if this heterosubtypic immunity can confer a level of human population immunity, preventing certain avian subtypes from becoming pandemic with less potential for promoting immune escape mutants [13] and on the role of such antibodies in the development of cross-protective human vaccines [14]. As the recent pandemics and avian influenza outbreaks demonstrated, there is a need to be better prepared to assess heterosubtypic and homosubtypic antibody responses to newly emerged viruses and to evaluate the extent of preexisting serological cross-reactivity in populations [15] as well as the lack of life-long immunity against influenza A viruses [10]. Additionally, from a public health point of view, it is important to determine if the immunological profiles of these responses can be detected using a combination of serological assays that can be subsequently used to promote vaccine efficacy testing and licensure. Influenza serological assays not only are routinely employed for virus characterization, vaccine strain selection, and vaccine evaluation but can also be exploited for assessing the composite nature of antibody responses generated against influenza A viruses, specific risk factors for infection, and rates of transmission in defined populations [2]. During the last decade, it has been approved by WHO and collaborating centers for influenza that studies on influenza vaccine efficacy should include endpoints that reflect a broad range of immune responses as a surrogate of protection, since protection represents the sum of various immune responses (including antibody and cell-mediated responses) and that there is the need to overcome limitations of the current available serological assays. To date, serum antibodies against the HA globular head are the only well-characterized and widely used correlates of protection while other correlates still need to be established for antibody responses against the HA stem regions, NA and M2 [16, 17]. The HI test is the most widely used assay due to its relative simplicity and although there is not an absolute titre that guarantees protection, the serum antibody titre of ≥1 : 40 remains associated with protection for inactivated influenza vaccines [18]. In the European community (EU) the SRH assay is also employed as an alternate serological assay for the evaluation of influenza vaccines [19, 20]. Undoubtedly, the need to develop effective pandemic vaccines and their evaluation in preclinical and clinical studies has raised questions. These primarily concern the evaluation of influenza vaccine immunogenicity and effectiveness in populations that are negative at baseline and in specific age groups (i.e., children and elderly people) [21, 22] or in sero-positive populations (before vaccination) where the same parameters may under- or overestimate the antibody responses [23]. Additionally, heterosubtypic responses can make interpretation of serological data more problematic. In light of this, international collaborative studies have been carried out not only to evaluate the reproducibility of current serological assays aiming to improve specificity, sensitivity, and interassay variability [20, 24] but also to reconsider certain criteria used for the evaluation of pandemic influenza vaccines which rely in essence on immunological endpoints that were derived for seasonal influenza vaccines [25].

In order to understand the contribution of broadly neutralizing “heterosubtypic” antibodies [26] and due to the initially observed lower sensitivity demonstrated for measuring anti-H5 antibody responses [27, 28] together with the requirements for working with highly pathogenic avian strains (Biosafety Level 3 (BSL3) laboratories), comparative serological studies have been carried out. These involve standard serological assays (SRH, HI, and MN assays) and the more recent pp-NT assay [28–31]. Influenza pseudotypes bearing HA and NA envelope glycoproteins devolved from the rest of the virus have been demonstrated to be ideal tools to monitor the effects of viral evolution on serological outcomes and can be used in parallel with other serological tests (HI, MN, and SRH), for accurate sequence-directed, sensitive, and low-containment assays for measuring antibody responses against influenza H5 and H7 HA and/or NA. It is relatively straightforward to update the pseudotype-based HA neutralization assay to measure responses against newly emerging H5 and H7 viruses [30, 32]. Pp-NT assays have been previously shown to have broad utility for the detection of neutralizing antibody responses in avian and human sera against both avian and human influenza viruses [31, 33, 34]. More specifically, pp-NT assays have been shown to be efficient for the measurement of broadly neutralizing antibodies directed against the HA stalk of influenza making them ideal serological tools for the study of cross-reactive responses against multiple influenza subtypes with pandemic potential [11, 35]. As described in the following study, influenza pseudotypes have been used to detect neutralizing antibody responses against HPAI H5 and H7 strains in human sera. These sera were found positive by SRH assay but the majority were negative when tested by well-established HI and MN assays, although this cohort of individuals has neither been vaccinated with a prepandemic H5N1 vaccine nor likely to have ever been exposed to H5N1 viruses [36].

## 2. Materials and Methods

### 2.1. Serum Samples

A panel of human sera was provided by Professor Emanuele Montomoli (Department of Molecular and Developmental Medicine, University of Siena, Italy), and this collection conformed to Italian ethics law. It consisted of 68 sera collected from Italian subjects (age between 6 months and 92 years old) from 1992 to 2007 that were previously found to be positive against HPAI H5N1 A/Vietnam/1194/2004 as determined by the SRH assay. The sero-positivity of these sera was also confirmed by removing nonspecific antibodies by their adsorption with a 1 : 1 volume mixture of A/New Caledonia/20/1999 H1N1 and A/California/7/2004 H3N2 viruses [36]. The positive controls used in pp-NT assays were reference sheep sera against A/England/427/1988 (H3N2) and NIBRG-14 (H5N1) provided by the National Institute for Biological Standards and Control (NIBSC, United Kingdom), avian sera against A/African starling/England/983/1979 (H7N1) provided by the Animal Health and Veterinary Laboratories Agency (AHVLA), and monoclonal antihuman influenza A (H1N1, H2N2) antibody (mAb C179) (Takara, Clonotech). The negative control used in the pp-NT assay was Foetal Bovine Serum.

### 2.2. Pseudotype Construction and Firefly Luciferase pp-NT Assays

Lentiviral pseudotypes with HA glycoproteins derived from HPAI virus A/Vietnam/1194/2004 (H5N1), A/Indonesia/5/2005 (H5N1), and HPAI virus A/chicken/Italy/13474/1999 (H7N1) were produced by cotransfection of 293T/17 cells with the respective HA plasmid (pI.18 backbone), the HIV gag-pol plasmid p8.91, and the reporter plasmid pCSFLW (expressing Firefly luciferase) using the Fugene-6 transfection reagent. Neuraminidase activity was provided by exogenous bacterial NA addition for the release of pseudotypes from producer cells. Serum samples (2.5 *μ*L) were twofold serially diluted in culture medium and mixed with pseudotype virus (1 × 10^6^ (RLU) luciferase input) at a 1 : 1 v/v ratio. After incubation at 37°C for 1 hr, 1 × 10^4^ 293T/17 cells were added to each well of a white 96-well flat-bottomed tissue culture plate. RLU were evaluated 48 hr later by luminometry using the Bright-Glo assay system (Promega, UK). End-point neutralizing antibody titres were calculated using GraphPad Prism 6. IC_50_ pseudotype neutralization titres are expressed as the reciprocal of the serum dilution that results in a 50% inhibition of pseudotype virus entry.

### 2.3. Single Radial Haemolysis (SRH) Assays

For the SRH assays, agarose immunoplates were prepared with sheep and turkey erythrocytes 10% (v/v of assay buffer) sensitized by inactivated whole virus (antigens used: A/Vietnam/1194/2004 and A/Indonesia/5/2005) and with the addition of 5% guinea-pig complement. The amount of antigen was diluted in PBS to reach a final concentration of 2000 HU/mL. The size of the haemolysis zone around the well containing the serum is measured in mm and the diameter of haemolysis is then transformed in area (mm^2^) by using a Transidyne Calibrating Viewer (Transidyne General Corporation, Ann Arbor, MI).

### 2.4. Inhibition of Haemagglutination (HI) Assays

The haemagglutination inhibition (HI) assay was performed according to WHO recommendation [37] and described previously [38] using whole inactivated virus for the H5N1 strains: A/Vietnam/1194/2004 and A/Indonesia/5/2005.

### 2.5. Microneutralization (MN) Assays

Microneutralization (MN) assays were performed as previously described [27, 39] using wild type H5N1 A/Indonesia/5/2005 (homologous strain of subclade 2.1 provided by CDC, Atlanta, GA) and wild type H5N1 A/Vietnam/1194/2004 (homologous strain of subclade 1 provided by the CDC).

## 3. Data and Statistical Analysis 

The end-point neutralizing antibody titres obtained by pp-NT assays were calculated and IC_50_ pseudotype neutralization titres were expressed as the reciprocal of the serum dilution that resulted in a 50% inhibition of pseudotype virus entry. For the panel of 68 sera (collected from Italian subjects between 1992 and 2007), the IC_50_ values obtained by pp-NT assay for different influenza strains were reported on Box-and-Whisker plots. When one-way ANOVA test was required, a nonparametric multicomparison one-way ANOVA was performed. Also, IC_50_ values obtained from pp-NT assays were reported on dot plot graphs and Pearson's correlation analysis was used to assess the correlation between pp-NT and HI titres, between pp-NT titres and SRH areas or between pp-NT titres and MN titres. All the analyses were performed by using Prism version 6 (GraphPad Software, Version 6, San Diego, CA). SRH results were considered negative in the absence of haemolysis or with an haemolysis area <4 mm^2^ (2.256 mm diameter), positive, but not sero-protective, with an haemolysis area between 4 mm^2^ and 25 mm^2^ (diameter between 2.25 mm and 5.65 mm), positive and sero-protective with haemolysis area ≥25 mm^2^ (diameter ≥ 5.65 mm). HI titres were defined as the reciprocal of the highest dilution resulting in complete inhibition of haemagglutination. HI titres were evaluated according to the EU EMEA CHMP criteria [40]. For MN assay, sera were tested at an initial dilution of 1 : 20 and those that yielded negative results were assigned a titre of 10.

## 4. Results 

### 4.1. HPAI H5 and H7 Influenza Pseudotype Production

For this study, lentiviral pseudotype particles were constructed as described previously [30, 32, 41] with the neuraminidase activity provided by exogenous bacterial NA addition. Pseudotype viruses were produced by cotransfection of human embryonic kidney (HEK) 293T/17 cells with the respective HA plasmids (HPAI H5N1 A/Vietnam/1194/2004 (clade 1), A/Indonesia/5/2005 (clade 2.1.3.2), and HPAI H7N1 A/chicken/Italy/13474/1999), the HIV* gag-pol* plasmid p8.91, and the lentiviral vector pCSFLW (expressing Firefly luciferase). A subset from the panel of 68 serum samples (with pp-NT titre range ≥320–640 when tested using H5N1 A/Vietnam/1194/2004) was additionally tested using influenza pseudotypes bearing the HAs from H3N2 A/Udorn/307/1972 and H1N1 A/South Carolina/1/1918.

### 4.2. Measurement of Neutralizing Antibodies by pp-NT Assays and Comparison between Serological Assays (Study Sera Collected from Italian Subjects between 1992 and 2007)

For each pseudotype tested (H5N1 A/Vietnam/1194/2004, A/Indonesia/5/2005, and H7N1 A/chicken/Italy/13474/1999), individual IC_50_ values were reported as titre ranges (from <40 to 5120–10240) (Figure 3). The H5 pseudotypes were completely neutralized by the H5 sheep sera (NIBRG-14 H5N1) at a 1 : 1280 dilution but not by the H7 antisera (A/African starling/England/983/1979) whilst the H7 pseudotype was completely neutralized by the H7 antisera at a 1 : 1280 dilution but not by the H5 sheep sera. In the absence of an appropriate sera control for H5 A/Indonesia/5/2005 and A/South Carolina/1/1918, C179 mAB was used, and H1 and H5 pseudotypes were neutralized with IC_50_ values ranging between 640–1280 and 1280–2560. Negative controls (Foetal Bovine Serum) did not show any neutralization.

The neutralizing antibody titres against the Group 1 H5N1 clade 1 A/Vietnam/1194/2004 pseudotype ranged from <40 (cutoff) to 1 : 5120–10240 and 7/68 (10.3%) sera were found to be negative while antibody titres against Group 1 H5N1 clade 2.1.3.2 A/Indonesia/5/2005 ranged from <40 to 640–1280 and 36/68 (53%) sera were found to be negative. IC_50_ neutralizing antibody titres against the Group 2 H7N1 A/chicken/Italy/13474/1999 pseudotype ranged from <40 to 1 : 160–320. 47/68 (69.1%) sera were found to be negative against A/chicken/Italy/13474/99 (Figure 3). Each individual serum sample tested against both H5 pseudotypes (subclades 1 and 2.1.3.2) and H7 pseudotype was also assigned a colour depending on the magnitude range (legend, Figure 3) in order to visualize the antibody titre range obtained for each serum sample against different influenza pseudotypes. The darker colours related to higher IC_50_ titres while lighter colours represented negative or lower neutralizing antibody responses and this allowed an immediate visualisation of the different antibody responses against HAs from HPAI H5 influenza pseudotypes compared to those obtained against HPAI H7 pseudotypes.

**Figure 1:**
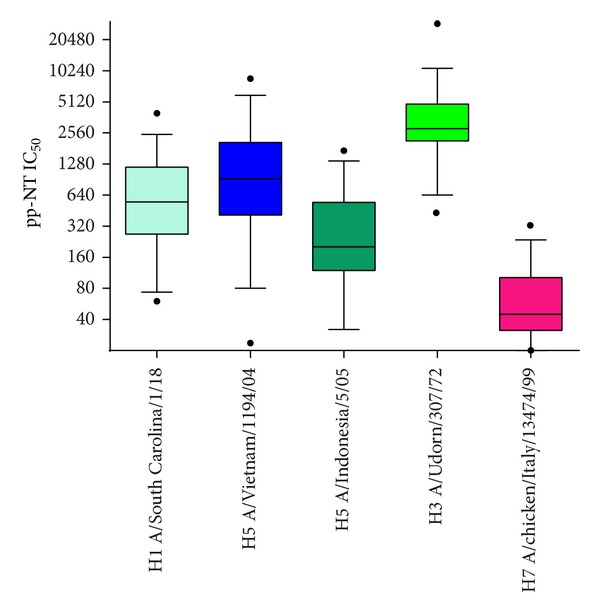
IC_50_ values (reported for each single virus as medians) obtained when 37 sera (with IC_50_ titres against H5 A/Vietnam/1194/04 H5 ≥ 320–640) were tested against H1, H5 (clade 1 and clade 2.1.3.2), H3, and H7 influenza pseudotypes and analysed by GraphPad using a nonparametric multicomparison one-way ANOVA test.

To explore the potential ability of influenza pseudotypes to detect heterosubtypic immune responses, neutralising antibody responses were evaluated not only against HPAI influenza pseudotypes but also against pseudotypes bearing HAs belonging to H1 and H3 subtypes. The individual IC_50_ titres of 37 sera (with pp-NT titres against A/Vietnam/1194/2004 H5 ≥320–640) were tested against Group 1 H1N1 A/South Carolina/1/1918 and Group 2 H3N2 A/Udorn/307/1972 and ranged from ≥40–80 to ≥2560–5120 and from ≥320–640 to ≥10240, respectively (Figure 4). All 37 sera resulted positive when tested by pp-NT assay. The IC_50_ values obtained for H1, H5 (from clades 1 and clade 2.3.1.2), H3, and H7 (expressed as medians and reported on Box-and-Whisker plots in Figure 1) were also analysed using nonparametric one-way ANOVA revealing no statistical differences between Group 1 influenza pseudotypes H1 A/South Carolina/1/1918 and H5 A/Vietnam/1194/2004 (*P* = 0.05), and H5 A/Indonesia/5/2005 (*P* = 0.05) and between H5 A/Indonesia/5/2005 and H7 (*P* = 0.05), while statistically significant differences were found between H1–H3 (*P* < 0.0001), H1–H7 (*P* < 0.0001), H5–H7, and H5–H3 pseudotypes (*P* < 0.0001).

With reference to SRH assay data, titres measured against the A/Vietnam/1194/2004 and A/Indonesia/5/2005 viruses ranged from 28.3 mm^2^ to 78.5 mm^2^ and from 3.9 mm^2^ to 78.5 mm^2^, respectively. All the subjects were classed as sero-protected against HA A/Vietnam/1194/2004 while 36 sera were scored with a value corresponding to ≤4 mm^2^; 11 subjects were positive but not sero-protected and 26 positive and sero-protected when tested against A/Indonesia/5/2005 (Table 1). By HI assay, excluding a single serum sample with a titre of 1 : 80 and two samples with a titre of 1 : 40, all other sera were found to be negative (HI titres ≤ 1 : 8, titre = 5) when tested against the A/Vietnam/1194/2004 virus. The HI results obtained for clade 2.1.3.2 H5 A/Indonesia/5/2005 mirrored those obtained by clade 1 H5 A/Vietnam/1194/2004 (Table 1). Similar results were found when the same panel of 68 sera was tested by MN assay (Table 1). All sera tested negative (MN titres = 7.01) against H5 A/Indonesia/5/2005 while a broad spectrum of antibody titres was detected against H5 A/Vietnam/1194/2004 although the majority of subjects were found negative or non sero-protected (MN titre ≤ 1 : 80), according to the proposed immune correlates of protection against H5N1 viruses for MN assays [25]. In detail, 7 subjects were found sero-protected (MN titres from ≥113 to 452.5), 16 were negative (MN titres ≤ 10), and 45 subjects had MN titres ranging between 14.1 and 56.5 (Table 1).

With all IC_50_ values (expressed as antibody titre range) the percentage of subjects (sero-responder) achieving a pp-NT titre ≥1 : 40, ≥1 : 80, ≥1 : 357 (which corresponds for pp-NT assay to the proposed threshold of protective antibodies (1 : 80) for MN assay) [30], and ≥20 were calculated and depending on these cutoff values, neutralizing antibody responses against different HAs were evaluated (Table 2). For a cutoff of 1 : 40, positive sera were found against H5 A/Vietnam/1194/2004 (61/68), A/Indonesia/5/2005 (42/68), and H7 A/chicken/Italy/13474/1999 (22/68). 37 sera, positive against H5 A/Vietnam/1194/2004 (titre ≥ 320–640), were also found positive against H3 (37/37) and H1 pseudotypes (37/37).

For a cutoff of 1 : 80: 56/68 sera were positive against H5 A/Vietnam/1194/2004, 30/68 against A/Indonesia/5/2005, 12/68 against H7 A/chicken/Italy/13474/1999, 37/37 against H3, and 34/37 positive against H1. When a cutoff 1 : 357 is chosen: positive sera were again detected against H5 A/Vietnam/1194/2004 (36/68) and 14/68 against A/Indonesia/5/2005 and all sera were found negative against H7 pseudotypes. Sera tested against H3 were all positive while 25/37 sera were positive against H1 pseudotypes when the same cutoff was used (1 : 357). Additionally, a further cutoff (≥20) was included based on a previous study where influenza pseudotypes have been employed for detecting heterosubtypic responses [14] and resulted in a higher number of positive sera against all strains: 67/68 for H5 A/Vietnam/1194/2004, 44/68 for A/Indonesia/5/2005, 53/68 for H7 A/chicken/Italy/13474/1999, and 37/37 for both H1 and H3 strains (Table 2).

**Figure 2:**
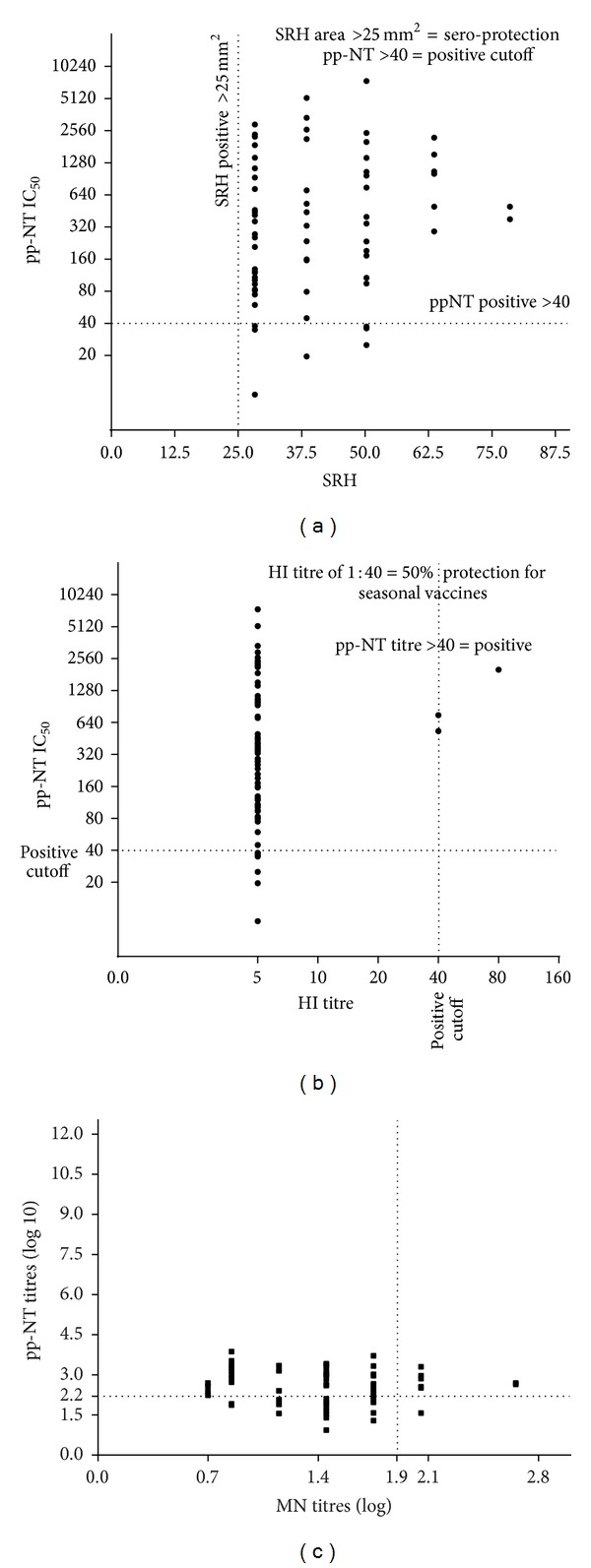
(a) Comparison of SRH assay versus pp-NT assay for antibody responses against the A/Vietnam/1194/04 antigen. SRH titres expressed as diameter of haemolysis (in mm^2^) are plotted on the *X*-axis. SRH titres > 25 mm^2^ are considered to be sero-protective and this cutoff is represented on the plot by a vertical dotted line. IC_50_ pseudotype neutralization titres expressed as the reciprocal of the serum dilution that results in a 50% inhibition of pseudotype virus entry are plotted on the *Y*-axis; (b) comparison of HI assay versus pp-NT assay for antibody responses against the A/Vietnam/1194/04 antigen. HI titres expressed as the reciprocal of the highest dilution causing complete inhibition of haemagglutination are plotted on the *X*-axis. IC_50_ pseudotype neutralization titres expressed as the reciprocal of the serum dilution that results in a 50% inhibition of pseudotype virus entry are plotted on the *Y*-axis; (c) comparison of MN assay versus pseudotype-based neutralization assay for antibody responses against the A/Vietnam/1194/04 antigen. The vertical dashed lines indicate the value of MN log 10 titre = 1.9 (corresponding to a titre of 1 : 80) and the proposed threshold of protective antibodies; horizontal dashed line indicates the corresponding value of pp-NT log 10 titre = 2.55 (corresponding to a titre of 1 : 357).

In order to assess whether the results obtained with the pp-NT assay reflected those obtained with conventional serological assays (using H5 A/Vietnam/1194/2004), data were compared to those obtained by HI, SRH, and MN assays (using the corresponding H5 A/Vietnam/1194/2004 strain). Pearson's correlation test was performed to measure the significance of the correlations with all data obtained from the panel of 68 sera. When results from pp-NT assay were compared to those obtained from SRH assay, no correlation was observed although both assays detected positive responses (SRH areas > 25 mm^2^ and pp-NT proposed cutoff > 40) (Figure 2); therefore, both assays were able to detect antibody responses against the analogous HPAI H5 strain.

Different serological results were obtained with pp-NT and HI assay where no correlation between assays was found but, unlike the situation with SRH, predominantly negative responses were detected as shown in Figure 2. Finally the result of the analysis (which compares pp-NT titres and MN titres) revealed no statistically significant correlation between antibody titres obtained with pp-NT and those obtained by MN (Figure 2). As described previously, the majority of sera were negative by MN compared to the positive responses detected by pp-NT assay.

## 5. Discussions and Conclusions

In this study, preexisting serological cross-reactivity against HPAI influenza viruses in human populations has been evaluated using a comparative serology approach. Initially, lentiviral pseudotypes have been used to investigate the neutralizing antibody responses against HPAI influenza viruses belonging to subtype H5 (clade 1 H5N1 A/Vietnam/1194/2004 and clade 2.1.3.2 H5N1 A/Indonesia/5/2005) and subtype H7 (H7N1 A/chicken/Italy/134734/1999) and were employed for the screening of human sera collected from 1992 to 2007 in the Italian population [36].

The use of H5 and H7 influenza pseudotypes allowed the measurement of neutralizing antibody responses against two antigenically distinct HPAI HAs (belonging to HA Group 1 and 2). The individual components employed for the construction of the pseudotypes used for this assay have been chosen from a set of interchangeable plasmids many of which we have used for serological assay development previously [32, 33]. Because this study aimed to look at the composite nature of neutralizing antibodies responses against different influenza HAs and possibly detect antibody responses against the HA globular head and HA stem region, the NA activity was provided by exogenous bacterial NA. Therefore, influenza pseudotypes expressing HA only employed in this study did not take into consideration the contribution of anti-NA antibody responses. Due to a logistical difficulty of obtaining a panel of true negative sera (from 1-year-old children, gathered by laboratory analyses in 1965) previously used for the SRH assay [36], hyper-immune sheep and avian sera raised against H5 and H7 viruses were employed as controls and they effectively neutralized cognate clade pseudotypes. This demonstrated a lack of antigenic cross-reactivity between these two subtypes being consistent with the HAs of H5 and H7 viruses belonging to two separate groups (Groups 1 and 2) and based on phylogenetic relationship analysis of currently described HA subtypes [42]. It was known that antibodies present in these hyper-immune sera were predominantly haemagglutination inhibition (HI) competent antibodies [43] that target the globular head of HA, thus inhibiting pseudotype entry into 293T/17 cells.

**Figure 3:**
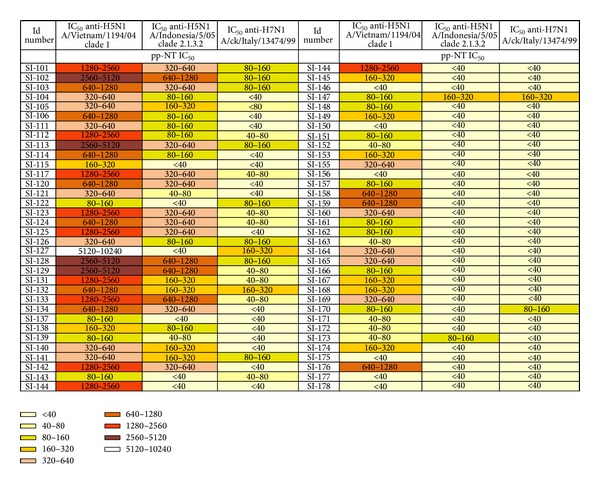
Cross-reactivity antibody profiles of SRH positive human sera against a Group 1 clade 1 H5 and clade 2.1.3.2 H5 and Group 2 H7N1 H7 pseudotypes as measured using pseudotype-based neutralization assays. Antibody response colour chart showing the IC_50_ neutralizing antibody titres of 68 sera with an SRH titre > 25 mm^2^ measured against H5 and H7 pseudotypes. Individual end-point titres (as calculated using GraphPad) were split into 9 separate bands based on titre range and assigned an individual colour. Colour legend is reported on the bottom of the figure.

**Figure 4:**
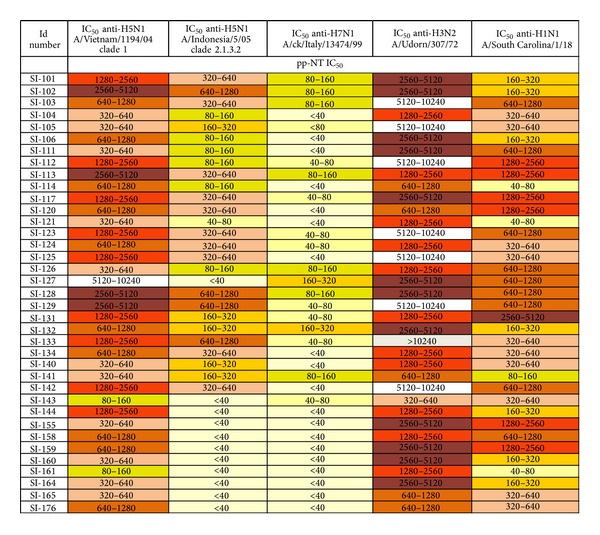
Cross-reactivity antibody profiles of SRH positive human sera against Group 1 H5 (A/Vietnam/1194/04, A/Indonesia/5/05) and H1 (A/SouthCarolina/1/18) and Group 2 H7 (A/chicken/Italy/13474/99) and H3 (A/Udorn/307/72) pseudotypes as measured using pseudotype-based neutralization assays. Antibody response colour chart showing the IC_50_ neutralizing antibody titres of 37 sera with an SRH titre > 25 mm^2^ measured against H1, H3, H5, and H7 pseudotypes. Individual end-point titres (as calculated using GraphPad) were split into 9 separate bands based on titre range and assigned an individual colour.

A comparative serology approach was undertaken by us for a prior study [30] where it was shown that in the context of a prepandemic vaccine trial, the pp-NT assay and the SRH assay exhibit significant correlation (*r* = 0.70, *n* = 226), which was also seen with HI (*r* = 0.73) and MN (*r* = 0.78). In order to determine if a similar degree of correlation can be obtained when these serological assays (SRH, pp-NT, HI, and MN assays) are employed for the study of antibody responses against A/Vietnam/1194/2004 in human subjects that have neither been vaccinated with a prepandemic H5N1 vaccine and that are unlikely to have ever been exposed to H5N1 viruses due to geographical locality (Italy), serum samples from Italian subjects that had previously been found positive by SRH against A/Vietnam/1194/2004 (titres > 25 mm^2^) were screened via pp-NT assay. Interestingly, the H5 antibody responses measured by the two assays in this context did not correlate as can be seen in Figure 2 where the plot showed that at each SRH point (28.3, 38.5, 50.3, 63.6, and 78.5 mm^2^) a wide range of pseudotype IC_50_ neutralization titres can be observed. As expected, similar results were observed when IC_50_ neutralization titres obtained when the same panel of sera was tested against HPAI H5 influenza pseudotypes belonging to different clade 2.1.2.3 (A/Indonesia/5/2005). Although a certain agreement was evidenced between SRH and pp-NT assays, the magnitude of antibody response was lower compared to that seen against H5 A/Vietnam/1194/2004 (as shown in Figures 3 and 4). Making direct comparisons between the two assays more complicated, SRH has been shown to detect not only antibody responses against the HA but also nonspecific antibody responses that are likely to have been elicited against NA and internal proteins such as NP and M [44, 45] whilst the pseudotypes used in this study do not have NA on their surface (unlike in the SRH) so any antibody responses detected using this assay are directed solely against the HA. However, results obtained when this panel of sera was tested using SRH and pp-NT assays suggested that a different mechanism of neutralization could be supported. In fact, when influenza pseudotypes were used, they have potentially detected antibody responses directed against epitopes not exposed on the HA globular head. Evidence for this was inferred by the marked absence of correlation when results obtained by pp-NT for both HPAI H5 strains were compared to those obtained by HI assay (Figure 2). The lack of correlation was further evidenced by the negative results obtained when the same panel of sera was used in an MN assay (Figure 2).

As previously shown, the detection of antibodies to avian influenza viruses in mammalian species using HI assay can be insensitive even in cases where experimental infection is confirmed by virus isolation [46, 47]. Therefore, the overall results of this comparative study could explain the extent of antibody responses obtained from the different serological assays (particularly HI and pp-NT). Considering the MN assay as the more sensitive test in detecting human antibodies to H5N1 virus in infected individuals [28], the total absence of antibody responses detected against A/Indonesia/5/2005 in this study cannot be simply justified by the assumption that the HAs on influenza pseudotypes are more accessible to neutralizing antibodies compared to the wild-type virus used in MN assay [31, 32]. Perhaps, one possible explanation could be sought in the MN method used in the reference laboratory as differences have been observed in other studies when virus neutralization was detected by ELISA in viral infected cells (using antibodies against NP), as opposed to the observation of viral cytopathic effect. Thus, aiming to further characterize the nature of these serological profiles obtained using the pp-NT assay, influenza pseudotypes bearing HA from HPAI H7 strains were also employed. The heterosubtypic antibody response as measured by this neutralization assay was found to be significantly higher against the Group 1 H5 pseudotype virus than against the Group 2 H7 pseudotype virus as shown in Figure 3. In 47/68 sera, no neutralizing antibody response was detected by the pseudotype assay against H7, and for the sera that were found positive against H7 titres were relatively low when tested by pp-NT. The assay described in this study consists of two serological antigens belonging to different HA Groups. The Group 1 subtype H5 shares a conserved HA stalk domain with H1, which also belongs to Group 1. The Group 2 subtype H7 shares a conserved HA stalk domain with H3. Both H1 and H3 viruses circulate continuously within the human population and are able to elicit broadly specific antistalk antibodies. Additionally, seasonal vaccination has been shown to elicit these antibodies [48] as well as primary infection in children [49]. As demonstrated by a precedent study where cross-reactive anti-avian H5N1 influenza neutralizing antibodies were found in normal “exposure-naïve” Australian blood donors [50], it is highly unlikely that the individuals sampled in this study had ever come into contact with HPAI H5 or H7 viruses or indeed antigenically related LPAI H5 or H7 viruses. Therefore, it is postulated that this highly sensitive neutralization assay is measuring broadly specific antistalk antibodies that have been elicited via exposure to H1 and H3 viruses and/or antibodies that recognize highly conserved sequences located underneath the RBS of individual subtypes (especially H1N1, H3N2, and H5N1) [51]. This is supported by the HI data, which shows that the responses measured are unlikely to be directed against the globular head. If this is indeed what is being measured, it suggests that H1 virus (Group 1) exposure is responsible for a more potent cross-reactive antibody response than H3 virus (Group 2) exposure. This can be explained by the fact that both H1 and H5 belong to the same Group 1 H1a Cluster (with H2 and H6), whereas H3 belongs to the H3 Cluster (with H4 and H14) and H7 belongs to the H7 Cluster (with H10 and H15) as described previously [10]. As the sera found sero-positive by SRH were also analyzed to remove nonspecific antibodies (these samples were adsorbed in a 1 : 1 volume mixture of A/New Caledonia/20/1999 H1N1 and A/California/7/2004 H3N2) and exclude any possible cross-reactivity [36], the serum samples found positive by pp-NT assay were tested not only against H5 and H7 subtypes but also against H1 (H1N1 A/South Carolina/1/1918) and H3 (H3N2 A/Udorn/307/1972) influenza pseudotypes so that additional information could be obtained from this panel of sera (Figure 4). As expressed by the percentage of positive sera (Figure 4), it was clear that the percentage of antibody positive responses against H1 and H3 pseudotypes were significantly higher than those seen for H5 and H7 (with 100% positive sera when tested against H3 independently of the positive cutoff chosen), and the neutralization pattern mirrored the phylogenetic relationship (similar magnitude of responses for H1 and H5 subtypes compared to H3 subtype). The antibody responses against H3 pseudotypes were significantly higher, however, previous studies have also demonstrated the presence of neutralizing activity against pandemic H1N1 and H5N1 pseudotyped viruses [34, 42]. Based on the serology data presented in this study, it is evident that further comparative serology studies are warranted to elucidate the nature of the heterosubtypic antibody responses elicited in these patients. Although results obtained by pp-NT assay correlated significantly with those obtained by HI and MN assays when testing avian sera [32] and the pp-NT assay was also reported to be more sensitive than classical MN when employed for the screening of sera from human subjects vaccinated against H5N1 [30], the positive threshold for pp-NT assays is still undefined and this makes it problematic to evaluate the reproducibility of different serological assays.

Since one of the purposes of this study was to understand if heterosubtypic antibody responses can be detected using influenza pseudotypes as serological tools as shown in a previous study [14], titres between 20 and 80 were taken into consideration. However, using the suggested positive cutoff for pp-NT assay of 1 : 357 [30] instead of those used to define a positive result or sero-protection by other serological assays, the serological outcome was noticeably changed for sera tested against HPAI H7 pseudotypes (Table 2). Although the majority of sera scored positive independently of the positive threshold used, the cutoff of 1 : 357 was unable to identify differentiated responses for sera tested by HPAI H7 pseudotypes. Therefore, this first study demonstrated that future refinement and further validation of this assay are warranted for the sero-epidemiological study of human populations. Moreover, additional aspects need to be taken into consideration such as identification of proper assay-specific cutoffs and internal/external controls, optimization of pseudotype titres, and availability of a more comprehensive library of influenza pseudotypes.

## Figures and Tables

**Table 1:** Panel of human sera tested by three different serological assays: SRH, HI and MN. For each assay, H5N1 strains belonging to clade 1 (A/Vietnam/1194/04) and clade 2.1.3.2 (A/Indonesia/5/05) were used. Anti-H5N1 responses were expressed as antibody titres for HI and MN assays while for SRH, the area of haemolysis (mm^2^) is reported.

Id number	SRH area mm^2^ anti-H5N1 A/Vietnam/1194/04	SRH area mm^2^ anti-H5N1 A/Indonesia/5/05	HI TITRE anti-H5N1 A/Vietnam/1194/04	HI TITRE anti-H5N1 A/Indonesia/5/05	MN TITRE anti-H5N1 A/Vietnam/1194/04	MN TITRE anti-H5N1 A/Indonesia/5/05
	SRH		HI		MN	
SI-101	28.274	28.274	5	5	7.071	7.071
SI-102	28.274	25.967	Serum not enough	Serum not enough	Serum not enough	7.071
SI-103	50.265	14.186	5	5	7.071	7.071
SI-104	28.274	28.274	5	5	452.548	7.071
SI-105	63.617	78.540	5	5	5.000	7.071
SI-106	63.617	50.265	5	5	56.569	7.071
SI-111	78.540	56.745	5	5	5.000	7.071
SI-112	50.265	44.179	5	5	28.284	7.071
SI-113	38.485	3.997	5	5	28.284	7.071
SI-115	50.265	12.566	5	5	5.000	7.071
SI-117	50.265	50.265	5	5	28.284	7.071
SI-120	63.617	12.566	5	5	452.548	7.071
SI-121	78.540	12.566	5	5	5.000	7.071
SI-122	50.265	25.967	5	5	56.569	7.071
SI-123	38.485	33.183	5	5	28.284	7.071
SI-124	28.274	12.566	5	5	14.142	7.071
SI-125	28.274	23.758	5	5	28.284	7.071
SI-126	38.485	9.621	5	5	7.071	7.071
SI-127	50.265	63.617	5	5	7.071	7.071
SI-128	38.485	44.179	5	5	56.569	7.071
SI-129	38.485	56.745	5	5	28.284	7.071
SI-131	63.617	3.997	5	5	28.284	7.071
SI-132	50.265	50.265	5	5	7.071	7.071
SI-133	28.274	33.183	5	5	28.284	7.071
SI-134	38.485	33.183	5	5	14.142	7.071
SI-137	28.274	3.997	Serum not enough	Serum not enough	Serum not enough	7.071
SI-138	28.274	38.485	Serum not enough	Serum not enough	Serum not enough	7.071
SI-139	38.485	56.745	Serum not enough	Serum not enough	56.569	7.071
SI-140	28.274	3.997	5	5	28.284	7.071
SI-141	50.265	3.997	5	5	7.071	7.071
SI-142	28.274	3.997	5	5	Serum not enough	7.071
SI-143	50.265	3.997	5	5	28.284	7.071
SI-144	63.617	3.997	5	5	56.569	7.071
SI-145	50.265	7.069	5	5	28.284	7.071
SI-146	28.274	44.179	5	5	56.569	7.071
SI-147	28.274	3.997	5	5	28.284	7.071
SI-148	28.274	3.997	5	5	56.569	7.071
SI-149	38.485	3.997	5	5	113.137	7.071
SI-150	50.265	3.997	5	5	56.569	7.071
SI-151	28.274	12.566	5	5	14.142	7.071
SI-152	38.485	3.997	5	5	14.142	7.071
SI-153	28.274	7.069	5	5	14.142	7.071
SI-155	38.485	12.566	40	5	7.071	7.071
SI-156	28.274	3.997	5	5	28.284	7.071
SI-157	28.274	3.997	5	5	14.142	7.071
SI-158	50.265	7.069	40	5	7.071	7.071
SI-159	28.274	12.566	5	5	56.569	7.071
SI-160	28.274	3.997	5	5	56.569	7.071
SI-161	50.265	3.997	5	5	28.284	7.071
SI-162	28.274	3.997	5	5	7.071	7.071
SI-163	28.274	3.997	5	5	28.284	7.071
SI-164	38.485	3.997	5	5	113.137	7.071
SI-165	28.274	63.617	5	5	113.137	7.071
SI-166	28.274	3.997	5	5	56.569	7.071
SI-167	28.274	3.997	5	5	56.569	7.071
SI-168	63.617	3.997	5	5	56.569	7.071
SI-169	50.265	3.997	5	5	56.569	7.071
SI-170	38.485	3.997	5	5	56.569	7.071
SI-171	28.274	3.997	5	5	7.071	7.071
SI-172	28.274	3.997	5	5	28.284	7.071
SI-173	38.485	3.997	5	5	28.284	7.071
SI-174	50.265	3.997	80	5	113.137	7.071
SI-175	50.265	3.997	5	5	28.284	7.071
SI-176	28.274	3.997	5	5	113.137	7.071
SI-177	38.485	3.997	5	5	56.569	7.071
SI-178	28.274	3.997	5	5	56.569	7.071

**Table 2:** Panel of 68 sera collected from Italian population between 1992 and 2007 and tested against H1, H5, H3, and H7 influenza pseudotypes. Number of positive sera was scored based on different positive thresholds (also expressed as a percentage).

	A/Vietnam/1194/04 H5N1	A/Indonesia/5/05 H5N1	A/ck/Italy/13474/99 H7N1	A/Udorn/307/72 H3N2	A/South Carolina/1/18 H1N1
1 : 40 cutoff*	61/68 ** (88%)**	42/68 ** (62%)**	22/68 **(32%)**	37/37 **(100%)**	37/37 **(100%)**
1 : 80 cutoff**	56/68 **82.3%**	30/68 **44.1%**	12/68 **17.6%**	37/37 **100%**	34/37 **92%**
1 : 357 cutoff***	36/68 **53%**	14/68 **20.5%**	All negative	37/37 **100%**	25/37 **67.5%**
≥20 cutoff****	67/68 **(98.5%)**	44/68 **(65%)**	53/68 **(78%)**	37/37 **100%**	37/37 **100%**

*HI positive cut-off which defines sero-protection in adults for seasonal vaccines [25].

**Proposed positive threshold for MN assay used to test human sera against HPAI strains [41].

***Corresponds for pp-NT assay to the proposed threshold of 1 : 80 for MN assay as previously shown [30].

****Positive cut-off suggested for the study of hetero-subtypic antibody responses by using influenza pseudotyped particles [14].
